# Supplementary material for: The Relationship between Binge Drinking and Binge Eating in Adolescence and Youth: A Systematic Review and Meta-Analysis
Source: Int J Environ Res Public Health. 2022 Dec 23;20(1):232. doi: 10.3390/ijerph20010232 (PMC9819376; doi:10.3390/ijerph20010232)
Supplement: Supplementary file 1 [file ijerph-20-00232-s001.zip › ijerph-2093856-supplementary.pdf]

Table S1. Studies selected for correlation coefficient

#	Study	Sample size	Age	Nationality	BE instrument	BD instrument	Correlation coefficient
1	Benjamin & Wulfert (2005) [115]	N = 335 (100% women)	Undergraduate students. Mean age: 19.6	USA	QEWP-R	Abbreviated version of the CORE Alcohol and Drug Survey	The frequencies of eating binges and alcohol use occasions were correlated *r* = 0.25, *p*<0.001
2	Cusack et al. (2021) [116]	N = 1,072 (100% women)	Undergraduate students. Mean age: 19.47	USA	EDE-Q Total Score	Above AUDIT cut-off	Partial correlation (binge eating - frequency of morning drinking) *r* = -0.02
3	Escrivá-Martínez et al. (2020) [25]	N = 428 (24,3% men and 75.7% women)	Undergraduate students. Mean age 21.67	Spain	Binge Eating Scale (BES)	Item 3 on the AUDIT scale (≥6 drinks on one occasion in the past year)	*r* = 0.226 (*p* < 0.001)
4	Horvath et al. (2021) [117]	N = 88 (100% men)	Mean age: 19	USA	EPSI binge eating score	AUDIT	Nonsignificant bivariate correlation between AUDIT and EPSI binge eating score (*r* = 0.19; *p* > 0.05).
5	Inguglia et al. (2019a) [118]	N = 944 (43% boys and 57% girls)	14-21. Mean age: 16.35	Italy	BES	AUDIT-3	*r* = 0.11; *p* < 0.01
6	Inguglia et al. (2019b) [119]	N = 570 (46% boys and 54% girls)	14-17. Mean age: 15.75	Italy	BES	AUDIT-3	*r* = 0.07; *p* > 0.05 (nonsignificant)
7	Laghi et al. (2012) [94]	N = 1,350 (47% boys and 53% girls)	17-19. Mean age: 17.46	Italy	BES. Score ≥18 "binge eaters"	≥5 drinks (≥4 females) on one occasion in the past month	Alcohol use frequency and binge eating were significantly correlated *r* = 0.31, *p* < .001.
8	Laghi et al. (2020) [87]	N = 1020 (50.6% boys and 49.4% girls)	16-22. Mean age: 17.9	Italy	BES. Score ≥17 "binge eaters"	≥5 drinks (≥4 females) on one occasion in the past 15 days and past month	Binge eating scale was correlated to binge drinking (*r* = 0.08; *p* < 0.05)
9	Martin et al. (2015) [120]	N = 153 (100% women)	Mean age: 19.72	USA	EDDS for Binge Eating Disorder (BED) symptoms	DDQ. ≥5 drinks in the past two weeks	Nonsignificant correlation between binge drinking and binge eating disorder *r* = - 0.13; p > 0.05
10	Rolland et al. (2017) [27]	N = 1,872 (42.6% men and 57.4% women)	20-23. Mean age: 21	France	Items 13, 14, and 15 of the EDE-Q, BE score	AUQ. BD score	Rho de Spearman *ρ* = 0.12; *p* < 0.001.

AUQ = Alcohol Use Questionnaire (AUQ); DDQ = Daily Drinking Questionnaire; EDDS = The 22-item Eating Disorder Diagnostic Scale; EDE-Q = Eating Disorder Examination Questionnaire; QEWP-R = Modified version of the 28-item Questionnaire on Eating and Weight Patterns-Revised

Table S2. Studies selected for odds ratio

#	Study	Sample size	Age	Nationality	BE instrument	BD instrument	Coefficient
1	Bui et al. (2021) [122]	N = 18,461 (48.5% boys and 51.5% girls)	11-18. Mean age: 14.9	Taiwanese	Emotional eating with two questions: In the past week, (1) how many times did you eat to reward yourself or celebrate? (2) How many times did you console yourself when feeling sad or stressed by eating?	>5 drinks on one occasion for at least one day during the past month	Subjects with binge drinking behavior were more likely to report frequent consumption of desert foods compared to participants who did not binge drink (OR = 1.24, 95% CI: 1.07–1.44).
2	Field et al. (2014) [39]	N = 5527 (100% boys)	12-18. Mean age: 14.9	USA	Binge eating at least 1/month and purged less than monthly (Binge Eating Disorder)	≥ 12 episodes of binge drinking per year	OR: 1.04 (0.55–1.98). 12-year prospective association between binge eating disorder and risk of developing binge drinking
3	Koupil et al. (2016) [123]	N = 12,599 (100% women)	18-23. Mean age: 20.8	Australia	DSM-IV: Binge eating (episodes of overeating with lost control)	Binge alcohol consumption was registered as 'at least once a week'	Relative risk ratios for alcohol binge drinking in a multivariate anallysis adjusted for age at start of follow-up of transient binge eating was 1.27 and 1.69 for persistent binge eating
4	Micali et al. (2015) [42]	N = 11,209 (42.9% boys and 57.1% girls)	Mean age: 14.57	UK	Binge Eating Disorder (BED) according to DSM-5 criteria	AUDIT-3	OR: 1.41 (0.68-2.93). Nonsignificant. BED Vs Binge drinking
5	Sonneville et al. (2013) [41]	N = 16,882 (46.5% boys and 53.5% girls)	9-14. Mean age: 11	UK	Binge Eating Disorder (BED) following DSM-5	Frequency cutoff of at least 6 episodes of binge drinking per year	Age and Sex-adjusted OR: 1.38 (1.06, 1.80). Fully adjusted OR: 1.14 (0.83, 1.57). Nonsingificant. Binge eating Vs frequent binge drinking. Binge eating was not associated with the onset of frequent binge drinking among either females (OR=1.15, 95% CI=0.83, 1.59) or males (OR=1.13, 95% CI=0.36, 3.55).
